# Effects of Frozen Stromal Vascular Fraction on the Survival of Cryopreserved Fat Tissue

**DOI:** 10.1007/s00266-019-01314-8

**Published:** 2019-02-15

**Authors:** Wanling Zheng, Jiawei Shen, Hao Wang, Yating Yin, Pingping Wang, Peisheng Jin, Aijun Zhang

**Affiliations:** 1 Department of Plastic Surgery, Xuzhou Medical University Affiliated Hospital, Xuzhou, China; 2 Department of Neurosurgery, Xuzhou Medical University Affiliated Hospital, Xuzhou, China; 3 Xuzhou Medical University, Xuzhou, China

**Keywords:** Cryopreservation, Fat tissue, Stromal vascular fraction, Fat grafts

## Abstract

**Background:**

Nowadays, the use of cryopreserved fat tissue for soft tissue augmentation is common, except for its unpredictable fat graft absorption, and the toxicity of the cryoprotective agent remains a limitation. In this study, the effects of freezing stored fat tissue without a cryoprotector, and the addition of the stromal vascular fraction (SVF) on the survival of cryopreserved transplants was studied.

**Methods:**

Lipoaspirates from six donors were processed and cryopreserved at − 20 °C, − 80 °C and − 196 °C, respectively. The authors evaluated the lipoaspirates in vitro, on the basis of fat tissue and SVF viability after cryopreservation. In vivo fat grafting was performed in nude mice. Six trenches were injected on the backs of mice. Cryopreserved tissues (− 20 °C, − 80 °C and − 196 °C) were injected on the right side, and the other side received the SVF combination. At 4 and 8 weeks after transplantation, the authors examined the weight, volume and morphology of the tissue and analyzed histochemical staining and immunohistochemistry (i.e., DIL, CD31 and VWF) to evaluate the survival of the fat grafts.

**Results:**

After cryopreservation without the cryoprotective agent, adipose tissue maintained its morphology better in − 80 °C than − 20 °C and − 196 °C. SVF cells can retain their adhesive and proliferative properties after cryopreservation. Although cryopreservation caused damage to fat tissue, all explants showed intact adipocytes and vascular ingrowth. Most of all, the − 80 °C group had less graft resorption and fibrosis than the other temperature groups. There was increased survival of fat grafts in the SVF group compared with the control group.

**Conclusion:**

In this study, the authors demonstrated that the storage temperature of − 80 °C was promising for 3 months of adipose tissue cryopreservation without a cryoprotective agent, and SVF could increase the survival rate of cryopreserved fat tissue.

**No Level Assigned:**

This journal requires that authors assign a level of evidence to each article. For a full description of these Evidence-Based Medicine ratings, please refer to the Table of Contents or the online Instructions to Authors www.springer.com/00266.

## Introduction

Autogenous fat grafts have been commonly used for soft tissue augmentation in plastic surgery, and their application is continuously expanding [1]. However, the survival rate of grafted fat only ranges from 30 to 80% [2, 3]. This problem might be attributed to some dead materials and insufficient revascularization in the grafts over time [4]. Moreover, partial resorption requiring repetitive injections can increase patient discomfort, morbidity, cost and time [5, 6]. To address these issues, cryopreservation of harvested autologous tissues is utilized.

An ideal cryoprotection solution should be nontoxic to cells and patients, nonantigenic, and chemically inert; provide a high survival rate after thawing; and allow for transplantation without washing [5, 7]. At present, no mature cryoprotectant for fat tissues has been developed, and the most commonly used cryoprotector, dimethyl sulfoxide (DMSO), shows cytotoxicity [8, 9]. Tissue cryopreservation requires a large cryoprotector dose, which cannot be permeated completely. In addition, this dose can lead to toxicity. Therefore, by using the cryoprotector in stromal vascular fraction (SVF) cryopreservation only, we can greatly reduce its dose and toxicity.

In this study, we investigated whether adipose tissue and SVF can survive after cryopreservation. Then, we determined the optimum storage temperature for adipose tissue cryopreservation. Finally, we investigated the effects of SVF on the survival of cryopreserved fat grafts. We first compared the viability of fat tissue at three common freezing temperatures: − 20 °C, − 80 °C and − 196 °C. Cryopreserved fat was thawed by bathing in 37 °C water for 5 min. The survival rates of cryopreserved fat were revealed by measuring the volume of the top layer separated after centrifugation [10], macroscopic and HE staining. Furthermore, we compared the proliferation properties of SVF after being cryopreserved in different temperatures and preparation processes. SVF cells from fresh adipose tissue, SVF cells from cryopreserved adipose tissue, and cryopreserved SVF cells were collected. The 3-(4,5-dimethyl-2-thiazolyl)-2,5-diphenyl-2-H-tetrazolium bromide (MTT, BD Bio-sciences, Franklin Lakes, NJ) assay was used to compare their adhesive and proliferative properties. The SVFs were mixed with cryopreserved fat tissue and injected into nude mice. The retained fat weight, volume and histology were evaluated.

## Materials and Methods

### Animal Model and Collection of Adipose Tissue

Adipose tissue was obtained from liposuction surgery from six healthy adult volunteers with a mean age of 30 ± 5.1 years and a mean body mass index of 25 ± 1.2 kg/m^2^. Patients who had systemic disorders or a history of chronic drug usage were not included in the study. The patients provided written informed consent, and the study was approved by the Institutional Review Board of Xuzhou Medical College Affiliated Hospital (Jiangsu, China).

Twenty-eight-week-old BALB/c-nu nude mice (Vital River, Beijing, China) weighing 20–23 g were used in the experiments. The animals were kept under controlled environmental conditions with constant laminar airflow, a temperature of 20–23 °C and humidity of 40–60%. They were given access to standard laboratory chow and sterilized water ad libitum. The Animal Care and Experiment Committee of Xuzhou Medical College (Jiangsu, China) approved the experimental protocol.

### Adipose Tissue Processing and Cryopreservation

Human aspirated fat tissue was obtained by liposuction and quickly transferred into a 50-mL tube, washed in phosphate buffered saline (PBS; Invitrogen, CA, USA) three times and centrifuged for 5 min at 1000 g. Upper and lower phases were removed. Then, the aspirated fat was aliquoted into 2 mL cryovials and frozen in different conditions: − 20 °C freezer, − 80 °C freezer and liquid nitrogen (frozen in a Nalgene Cryo 1 °C freezing container and placed in a − 20 °C freezer for 4 h with controlled rate of freezing of − 1 °C/min and transferred to a − 80 °C freezer overnight). All adipose tissues were frozen 3 months before analysis, and twenty vials of adipose tissue were frozen in each temperature.

### Preparation of SVF from Human Fat Tissue

To prepare SVF cells, subcutaneous adipose tissues were digested for 50 min at 37 °C (0.1% collagenase type I; Invitrogen, CA, USA), filtered, centrifuged and then cryopreserved. To compare the differences in the proliferative capacity of the three groups of SVF cells (SVF cells from fresh adipose tissue, SVF from cryopreserved adipose tissue and cryopreserved SVF), the pellet was resuspended in Dulbecco’s Modified Eagle’s Medium (DMEM; Invitrogen, CA, USA) containing 10% fetal bovine serum (FBS; Invitrogen, CA, USA) and placed in a 37 °C humidified incubator with 5% CO_2_. The cells were maintained for 4–5 days until confluent. All SVFs were obtained from a single donor.

SVFs were labeled with 1,1′-dioctadecyl-3,3,3′,3′-tetramethylindocarbocyanine (CM-Dil, CA, USA) prior to injection. Briefly, cells in suspension were incubated with CM-Dil at a concentration of 5 μg/mL in PBS for 10 min at 37 °C and then for 15 min at 4 °C. They were then washed with PBS three times. The efficiency of CM-Dil staining was detected with a fluorescence microscope.

### Viability of Adipose Tissue and SVF after Cryopreservation

Tissue was thawed in a water bath at 37 °C after cryopreservation. The degree of tissue damage was evaluated by measuring the volume of the supernatant lipid layer after centrifugation at 1000 rpm for 5 min. The morphology of cryopreserved tissue was assessed by HE staining. In addition, the viability of adipocytes was evaluated by trypan blue exclusion. The number of live adipocytes after freezing at 4 °C, − 20 °C and − 80 °C without cryoprotector can be used to reflect the viability of cryopreserved fat tissue indirectly.

The proliferation of SVF was analyzed by using MTT and CellQuest software. SVF cells from fresh adipose tissue, cryopreserved SVF and SVF cells from cryopreserved adipose tissue were plated at a density of 1 × 10^4^ cells/well in 96-well plates. After washing, a culture medium containing 0.5 mg/mL MTT was added to each well. The cells were then incubated for 4 h at 37 °C. Afterward, the supernatant was removed, and the formazan crystals that had formed in the viable cells were solubilized using 150 μL DMSO. The absorbance was measured at 490 nm using a microplate reader. This experiment was repeated three times.

### Animal Model: Fat Graft in Nude Mice

The mice were anesthetized using 4% chloral hydrate. Cryopreserved adipose tissues were injected on the back of mice on either side of the vertebral column. We injected 0.1 mL adipose tissue for each trench. Mice received − 20 °C, − 80 °C and − 196 °C cryopreserved tissues on one side, and a mixture containing 0.1 mL cryopreserved fat tissue and 20 μL PBS containing 1 × 10^6^ SVF on the other side. Trenches were homogeneous, measuring 1 cm long and 1 cm wide. Each animal received six fat grafts from the same donor, totaling 36 specimens of cryopreserved adipose tissue grafts and 36 specimens of SVF-treated cryopreserved adipose tissue grafts.

### Follow-up and Data Collection

The length, width and height of the trenches were measured twice a week to assess the kinetics of the area. Two mice were humanely sacrificed after 1 month to observe whether SVFs were viable and functioned. Then, the grafts were carefully dissected from the back of mice after euthanasia. CM-Dil-labeled SVFs were observed under a fluorescence microscope with excitation at 420 nm and emission at 480 nm [11] (Olympus, Tokyo, Japan).

The fat grafts were collected at 4 and 8 weeks. Their volume and weight were measured to assess the percentage of resorption. The volume was determined using the liquid overflow method. (The graft was placed in a 1 mL container filled with water, then the volume of the overflow water was measured by microliter syringe, and this was the volume of the graft.) Then, fat specimens were fixed in 10% formalin and embedded in paraffin. Sections (5 μm) were stained with HE for light microscopy and incubated with CD31 (1:160), VWF (1:160) and collagen (1:50) antibody (Cell Signaling Technology, USA) and diaminobenzidine. Images were obtained using a Camedia Master C-3040 digital camera. Histological evaluations were performed by a professional pathologist in a blinded manner. Each slide was evaluated for (1) the average number of intact adipocytes, cysts and vacuoles; (2) rate of fibrosis, inflammation and other components of connective tissue; and (3) angiogenesis.

### Statistical Analysis

The Mann–Whitney rank-sum test was used to compare the volume and weight of fat and fat tissue histological parameters among the groups. IBM SPSS version 20.0 (IBM Corp., Armonk, NY, USA) for Windows was used for statistical analysis. For differences between the SVF-treated and control groups, *p* < 0.05 was considered to indicate statistical significance.

## Results

### Viability of Adipose Tissue After Cryopreservation

**Fig. 1:**
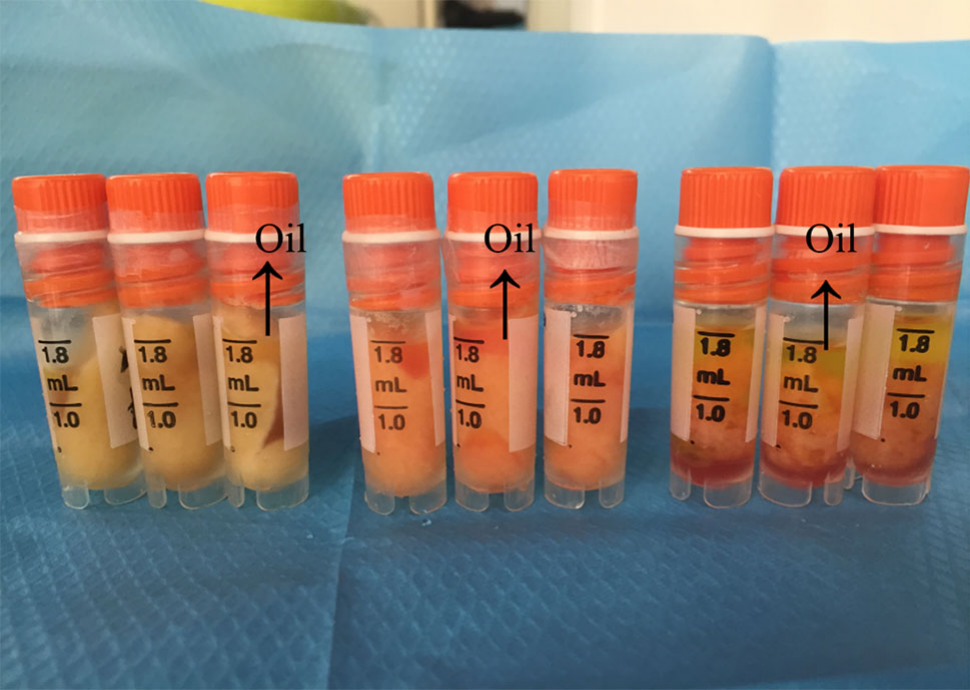
Macroscopic appearance of the cryopreserved fat tissue after thawing. The − 20 °C cryopreserved fat (left), − 80 °C cryopreserved fat (middle) and − 196 °C cryopreserved fat (right)

To determine the extent of adipocyte death caused by cryopreservation, we measured the volume of oil released from adipose tissue treated under different cryopreservation conditions. Figure 1 shows that at a lower temperature (from − 20 °C to − 196 °C), more oil was released from the tissue (5–90%). This finding demonstrated that cryopreservation at − 196 °C without a cryoprotector was extremely harmful for adipose tissue. Compared with that at − 20 °C, cryopreservation at − 80 °C resulted in the release of more oil from the adipose tissue, but the difference was not significant (85 vs. 90%, respectively). Moreover, the grafts cryopreserved at − 20 °C were dark and gloomy.

**Fig. 2:**
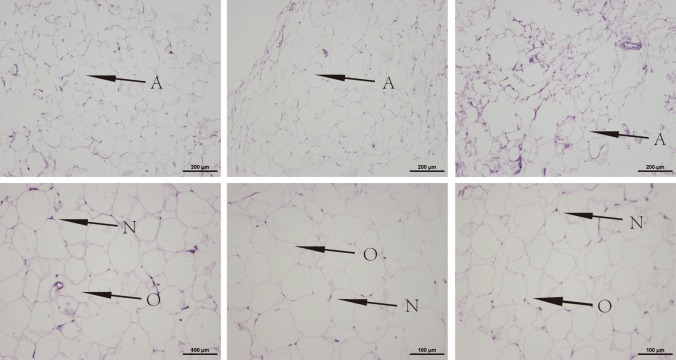
Histological structure of the cryopreserved fat tissue. The − 20 °C cryopreserved fat (left), − 80 °C cryopreserved fat (middle) and − 196 °C cryopreserved fat (right). Magnification 100X (above) and 200X (down). A: adipocyte; O: oil lacuna; N: nucleus

HE staining was performed after rapidly thawing the cryopreserved adipose tissues. Figure 2 shows that all cryopreserved adipocytes maintained a basic structure and that at lower temperature, more fibrous connective tissues and oil lacuna were retained. The adipocytes cryopreserved at − 20 °C showed many impurities. By contrast, the adipocytes cryopreserved at − 80 °C showed uniform size and close arrangement and maintained better morphology.

### Viability of SVFs after Cryopreservation

**Fig. 3:**
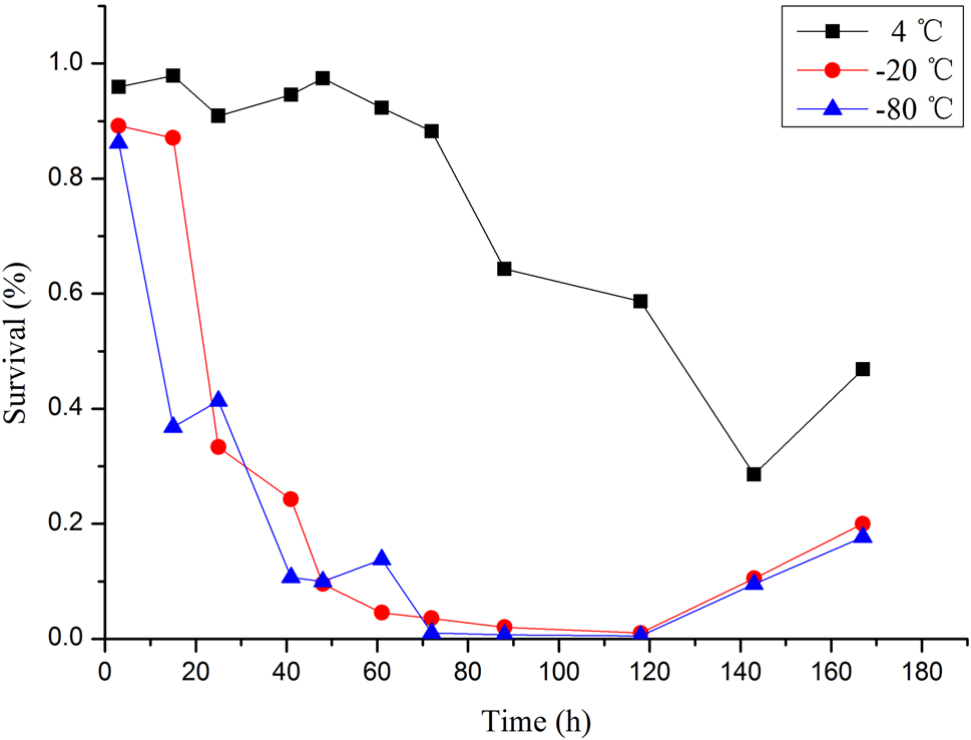
Comparisons of three freezing temperatures on SVF. The − 20 °C cryopreserved fat (red line), − 80 °C cryopreserved fat (blue line) and 4 °C cryopreserved fat (black line)

**Fig. 4:**
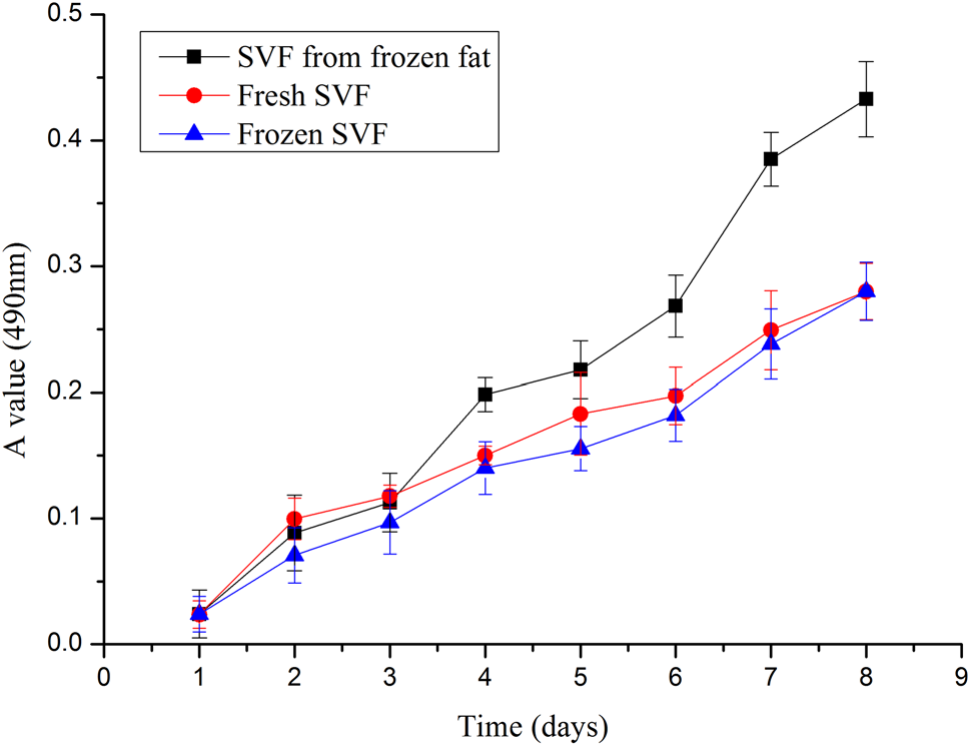
The capacity of SVF to proliferate was tested by MTT, with comparison of SVF cells from fresh adipose tissue (red line), cryopreserved SVF (blue line) and SVF cells from cryopreserved adipose tissue (black line)

The damage to the SVF was evaluated by counting the number of viable cells through trypan blue staining. Without a cryoprotective agent, the number of living SVFs at − 20 °C and − 80 °C cryopreservation temperatures were very low, even after 1 day of storage (Fig. 3). After 1 week, we attempted to isolate SVF cells from cryopreserved adipose tissue without a cryoprotective agent. However, the cells could only be isolated from fat tissue cryopreserved at 4 °C. This result indicates that 4 °C cryopreservation temperature is beneficial for short-term storage. Although the number of SVF cells derived from equivalent amounts of frozen fat is small, the proliferation activity of such cells is good. Compared with fresh adipose tissue SVF, the cryopreserved adipose tissue SVF without a cryoprotector showed a nearly twofold increase in number in the MTT assay. This result indicates that cells with better proliferation ability may be retained and those with poorer proliferation ability may be eliminated during cryopreservation. No significant difference was found between the proliferation of fresh SVF and cryopreserved SVF with a cryoprotective agent. Adherent cells displayed similar fibroblastic morphology, and the proliferative potential of SVF was not compromised by cryopreservation (Fig. 4).

### Fat Tissue Survival

**Fig. 5:**
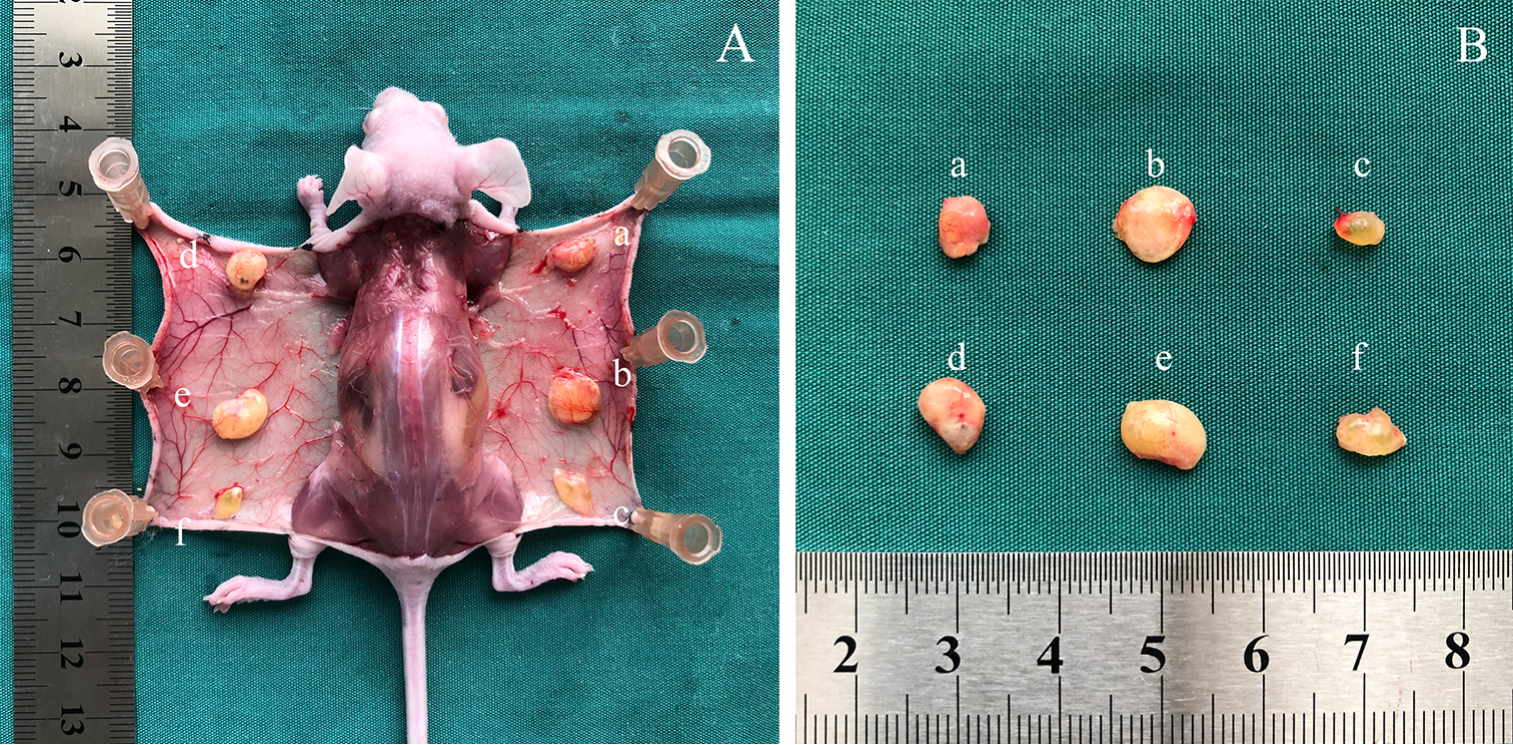
(Left) Fat grafts after transplantation: the samples were harvested four and 8 weeks after the fat grafting procedure. (Right) Macroscopic aspect of harvested fat grafts: control group (above) and SVF-treated group (down). **a**, **d** − 20 °C group; **b, e** − 80 °C group; and **c**, **f** − 196 °C group

**Fig. 6:**
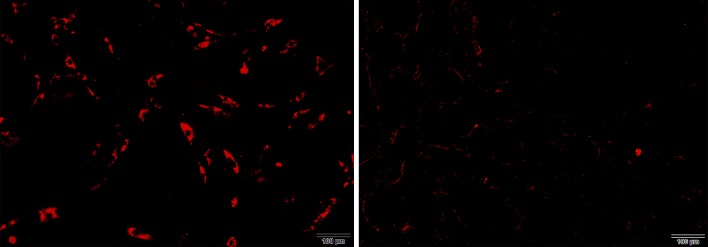
CM-Dil labeling of SVF cells in vitro and in vivo. The components derived from Dil-labeled SVF cells exhibit red fluorescence. All images were taken at 200 magnification. (Left) Before transplantation. (Right) After transplantation

After transplantation, all mice survived with soft skin and good filling effect. A thin layer of film formed around the graft areas, making them easy to separate. Peripheral vascularization was noticed, although some oil cysts were evident (Fig. 5a). The dissected fat grafts were weaker and more fragile in the control group than in the SVF-treated group, and those in the − 196 °C-treated group were nearly all oils (Fig. 5b). The survival of the transplanted SVF was demonstrated by the presence of red-fluorescing CM-Dil-labeled cells. Some red-fluorescing cells were detected around mature adipose tissues, which indicate that some of the adipocytes in the tissue were derived from the exogenous SVF (Fig. 6).

### Analysis of the Volume of Transplanted Fat

The animals were selected at random and sacrificed at 4 and 8 weeks after transplantation. The weight and volume of the remaining grafted fat tissue were determined at each time point. A significantly higher survival of cryopreserved fat grafts was observed in the group treated with SVF compared with the control group (*p* < 0.05). Moreover, a difference of resorption was noted among the three temperatures, and a significantly higher survival of cryopreserved fat grafts was observed in the − 80 °C-treated group than in other groups (*p* < 0.05) (Table 1).

**Table 1:** The amount of fat tissue harvested from the mice

Group(ml)	Fat frozen in − 20 °C		Fat frozen in − 80 °C		Fat frozen in − 196 °C	
	4 weeks	8 weeks	4 weeks	8 weeks	4 weeks	8 weeks
Control	0.057±0.010	0.039±0.010	0.068±0.010	0.059±0.011	0.033±0.016	0.032±0.022
SVF	0.070 ± 0.011^*^	0.056 ± 0.010^*^	0.080 ± 0.009^*^	0.083 ± 0.008^*^	0.041 ± 0.017^*^	0.051 ± 0.020^*^

^*^p < 0.05 compared with control group

### Histological Evaluation and Immunohistochemistry

**Fig. 7:**
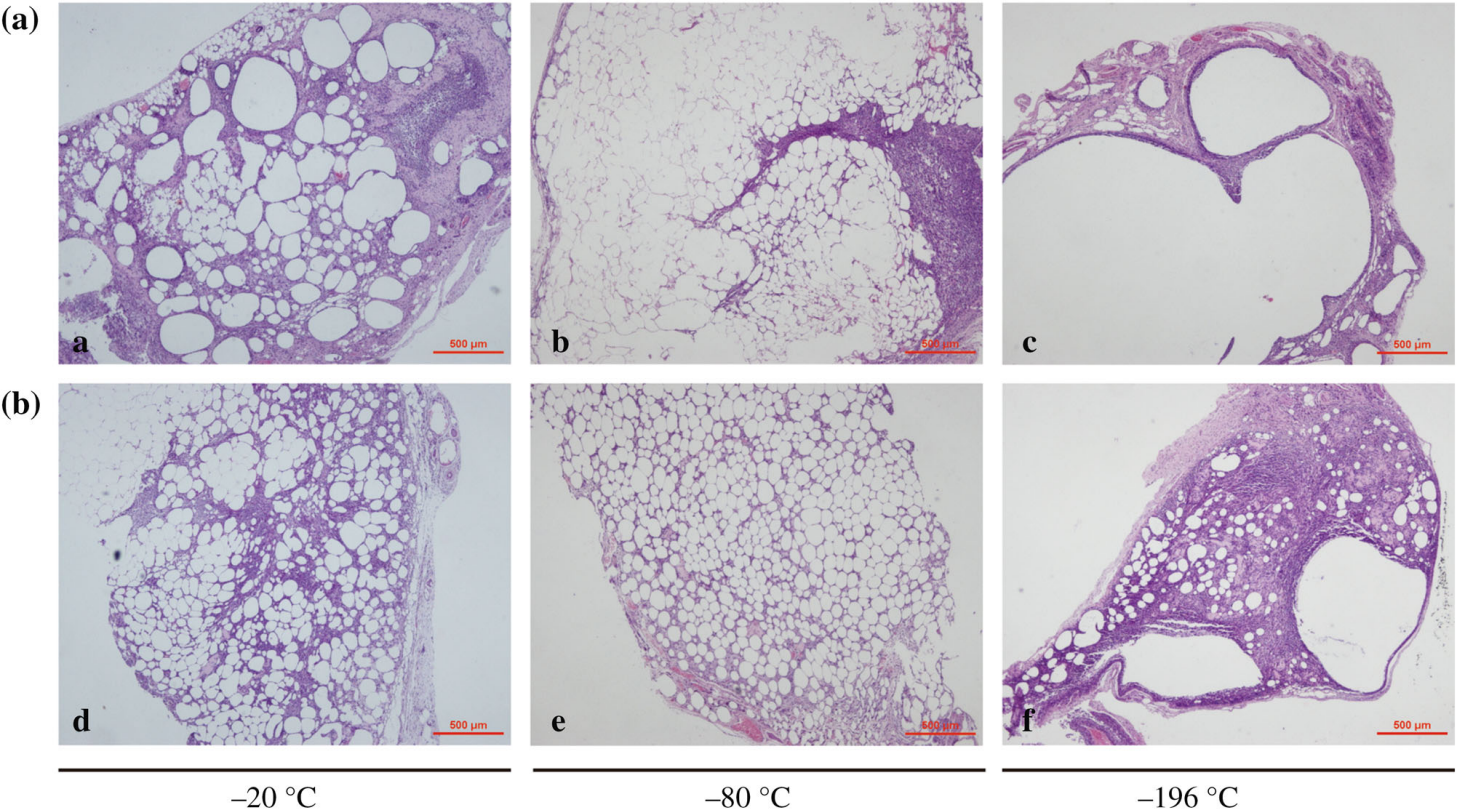
Histological features of fat grafts after transplantation. All images were taken at 40 magnification. **a** Control group and **b** SVF-treated group

**Fig. 8:**
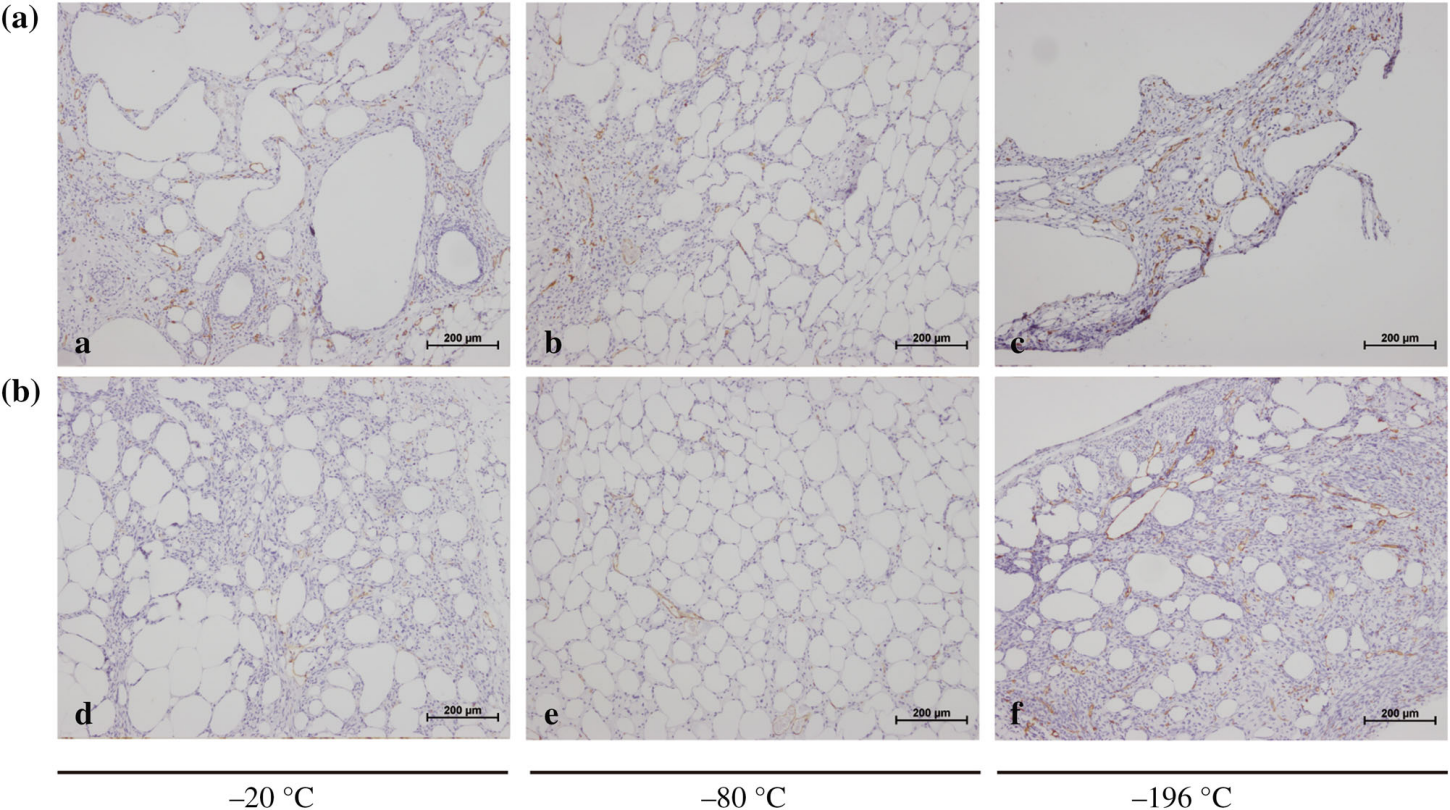
Immunohistochemical staining for CD31 was performed to evaluate the effect of SVF on cryopreserved fat grafts. CD31 + endothelial cells exhibit brown coloration. All images were taken at 100 magnification. **a** Control group and **b** SVF-treated group

**Fig. 9:**
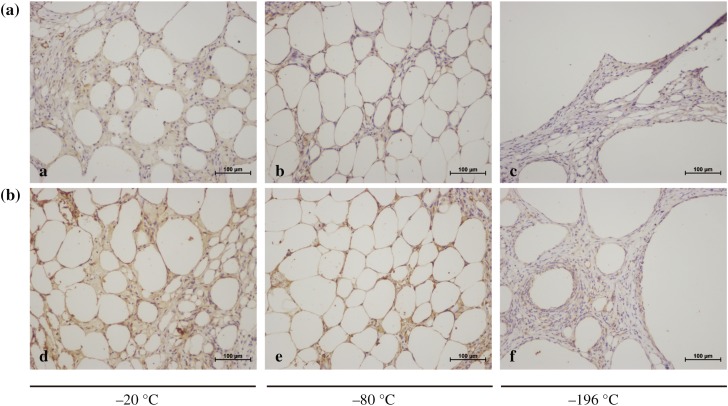
Immunohistochemical staining for VWF was performed to evaluate the effect of SVF on cryopreserved fat grafts. VWF + endothelial cells exhibit brown coloration. All images were taken at 200 magnification. **a** Control group and **b** SVF-treated group

After transplantation, histological evaluations were performed by staining fat grafts with HE. The results showed that the SVF-treated group had better fat cell integrity and less inflammation and fibrosis than the control group (Fig. 7). Among the groups, the fat grafts cryopreserved at − 20 °C produced excessive fibrosis and inflammation, and those cryopreserved at − 196 °C had evident cysts and vacuole formation. CD31 and VWF immunohistochemical staining of fat graft vasculature demonstrated greater uptake in SVF-added grafts compared with the control group (Figs. 8, 9). The differences among the groups were all statistically significant (Table 2).

**Table 2:** Effects of SVF on fat graft histological parameters (*n* = 6**)**

Group	Intact cells			Fibrosis			Vascularization		
	− 20 °C	− 80 °C	− 196 °C	− 20 °C (%)	− 80 °C (%)	− 196 °C	− 20 °C	− 80 °C	− 196 °C
Control	18.7 ± 2.2	43.8 ± 2.6	4.3 ± 1.8	24.8 ± 1.0	14.2 ± 2.3	17.3 ± 1.3	21.1 ± 1.8	31.0 ± 1.5	20.0 ± 0.8
SVF	42.8 ± 4.6^b^	69.7 ± 10.5^a^	8.0 ± 1.4^a^	19.1 ± 1.4^b^	10.4 ± 1.1^a^	15.9 ± 0.6^a^	24.8 ± 2.0^a^	33.6 ± 2.1^a^	22.5 ± 1.8^a^

Data are expressed as mean ± SD

^a^*p* < 0.05 compared with control group

^b^*p* < 0.001 compared with control group

## Discussion

Although liposuction is a simple procedure for harvesting adipose tissues, the repetition of this surgical intervention can cause adverse effects including higher cost, unfavorable appearance, increasing patient morbidity, discomfort and can be a limiting factor for immediate use [5, 12, 13]. Cryopreservation can avoid the morbidity associated with repetitive liposuction, allowing the use of stored tissue after the initial harvest procedure. Currently, the standard temperature for adipose tissue cryopreservation is not yet known, and no mature study has been conducted to determine the survival mechanism after fat transplantation. In the present study, we developed a cryopreservation protocol that could be applied directly without a cryoprotector (DMSO).

In our study, the viability of adipose tissues was difficult to maintain when they were cryopreserved at − 20 °C for 3 months without a cryoprotective agent. The tissues showed a dark color. Moreover, their volume decreased significantly when they were cryopreserved in liquid nitrogen. Thus, achieving a good transplantation effect was difficult as well. Two months after transplantation, all nude mice survived, and the skin on their back was velvety. The surface of grafts was completely covered with a thin layer of film, and peripheral vascularization was noticed. A significantly higher survival of cryopreserved fat grafts and more angiogenesis were observed in the group treated with SVF than in the control group. Moreover, the grafts cryopreserved at − 80 °C maintained better color and volume and intact adipocytes than those cryopreserved at other temperatures. Among the groups, the grafts cryopreserved at − 20 °C had progressive fibrosis and inflammation, and several oil cysts were found in grafts cryopreserved in liquid nitrogen. This study demonstrated that compared with other temperatures, -80 °C was more suitable for fat tissue cryopreservation when freezing for 3 months without a cryoprotector, and the survival rate of tissues could be increased with the use of SVF. In addition, SVF cells from fresh adipose tissue, cryopreserved SVF and SVF cells from cryopreserved adipose tissue all displayed similar fibroblastic morphology, and the proliferative potential of SVF was not compromised by cryopreservation. Previous studies have observed no significant effects on differentiation potential after freezing [14–17], indicating that SVF can be stored for a long time with a cryoprotector.

Fat tissues can survive after transplantation, but the mechanism underlying the survival of transplanted fat tissues remains unclear. Currently, three theories (substitution theory, survival theory and preadipocyte theory) are used to explain this mechanism. Through immunofluorescence labeling, we found that the exogenous SVF can transform into adipocytes in frozen fat grafts. Thus, SVF can directly improve the survival of frozen grafts. Zanata [18] transplanted cryopreserved fat tissue onto the back of green fluorescent protein mice and found positive staining for green fluorescent protein cells after removing the graft, indicating that the frozen grafts survived partly from the receptor.

In the present study, fat tissues cryopreserved for 3 months without a protective agent also achieved a good filling effect. This finding may be attributed to the following: First, cryopreserved fat tissue is not only a dead filler. Some stem cells and capillary cells surrounding this tissue may have survived or become dormant temporarily, which is similar to the encapsulation and preservation of hydrogels made by Zhao [19, 20]. With controlled cooling, stem cells and endothelial cells can be protected and retain their activity. Second, frozen fat can be used as an effective extracellular matrix. Glycoproteins, cytokines and albumin in the cryopreserved tissue can be retained even if no living cells are present under freezing without a cryoprotector. These components can support the growth of surrounding cells. In a recent study, Lu [21] prepared a vascular matrix gel by destroying normal adipose tissue and found that this graft without mature adipose cells exhibited a good filling effect and the adipocytes could survive. As more oils can be released after long-term cryopreservation without a cryoprotector, the components retained after removing the inactive grease through centrifugation can be used as a skeleton. This skeleton is similar to the matrix scaffold gel and can recruit local preadipocytes from the host to stimulate endogenous adipose regeneration. Thus, the retained components can maintain the volume, promote the production of new adipocytes and improve the survival of frozen grafts.

Our findings can change the way we treat our patients undergoing fat grafting. They can serve as a basis for developing a solution to the need of repeated liposuctions for lipofilling treatments and for establishing a suitable cryopreservation method. In addition, the excess fat can be extracted and stored as SVF to assist with secondary operations.

Despite our findings, this study has some limitations. For example, the number of specimens is relatively small. We did not distinguish between fat graft retention and endogenous adipose regeneration. To explore the source of filling effect of long-term frozen adipose tissue transplantation, fluorescent labeling of frozen adipose tissue will be conducted in subsequent studies. In addition, we only used frozen fat and SVF cryopreserved for 3 months. The optimum cryopreservation temperature may change in the future. Moreover, an ideal protective agent still needs to be developed.

## Conclusion

We found that frozen fat can partly survive after transplantation without a protective agent. The storage temperature of − 80 °C is promising for 3 months of adipose tissue cryopreservation, and SVF can increase the survival rate of fat grafts.
